# Detection of Abalone Freshness Based on Smart Phone Image and Deep Learning

**DOI:** 10.3390/foods15183189

**Published:** 2026-09-09

**Authors:** Yizhan Yu, Jialin Li, Junlong Lai, Zhihong Zheng, Haisheng Lin, Wenhong Cao, Jialong Gao, Xiaoyu Xia

**Affiliations:** 1 Guangdong Provincial Key Laboratory of Aquatic Products Processing and Safety, National Research and Development Branch Center for Shellfish Processing (Zhanjiang), College of Food Science and Technology, Guangdong Ocean University, Zhanjiang 524088, China; 18933270920@stu.gdou.edu.cn (Y.Y.); ljl113@stu.gdou.edu.cn (J.L.); ljl214@stu.gdou.edu.cn (J.L.); zhzheng617@163.com (Z.Z.); linhs@gdou.edu.cn (H.L.); cwenhong@gdou.edu.cn (W.C.); gaojl@gdou.edu.cn (J.G.); 2 Collaborative Innovation Center of Seafood Deep Processing, Dalian Polytechnic University, Dalian 116034, China

**Keywords:** abalone, freshness, smartphone imaging, deep learning, ResNet50

## Abstract

Rapid and nondestructive freshness evaluation of abalone is important for quality control during cold-chain distribution, yet conventional chemical and microbiological methods are destructive and labor-intensive. In this study, a smartphone image-based deep learning strategy was developed for abalone freshness classification under refrigerated storage. Abalone samples stored at 4 °C were imaged daily under natural light, and freshness labels were assigned according to total volatile basic nitrogen (TVB-N) measurements. A total of 1867 images were used to develop binary classification models, and a transfer learning-based ResNet50 model was further interpreted using Gradient-weighted Class Activation Mapping (Grad-CAM). TVB-N increased progressively during storage and exceeded the spoilage threshold on day 5 (15.63 ± 0.43 mg/100 g), which was used to define fresh (days 1–4) and spoiled (days 5–7) classes. Among the evaluated architectures, ResNet50 achieved the best overall performance, with a validation accuracy of 0.9611, precision of 0.9649, recall of 0.9091, and F1-score of 0.9362. On the test set, the model correctly classified 522 fresh and 220 spoiled images, yielding an overall accuracy of 96.11%. Grad-CAM visualization showed that the model mainly focused on the abalone body and marginal contour, indicating that predictions were driven by intrinsic appearance changes rather than background interference. These results demonstrate that smartphone imaging combined with deep learning provides a rapid, low-cost, and nondestructive approach for abalone freshness assessment and has potential for digital quality monitoring in shellfish cold chains.

## 1. Introduction

With the expansion of cold-chain distribution and consumer demand for high-value seafood, maintaining the freshness of abalone during circulation has become increasingly important. Abalone is valued not only for its high nutritional and economic significance, but also because its quality status is directly associated with consumer food safety and the commercial value of downstream products across the supply chain. Therefore, the establishment of a rapid, accurate, and distribution-oriented freshness evaluation method is of great importance for ensuring the quality and safety of abalone products and improving supply chain management efficiency.

At present, the evaluation of abalone freshness still mainly relies on methods such as total volatile basic nitrogen (TVB-N), total viable count, and sensory assessment [1]. Among these, TVB-N has become a commonly used chemical indicator in aquatic product quality evaluation because it can effectively reflect the extent of protein degradation and spoilage [1,2]. However, these methods usually involve sampling, homogenization, distillation, and titration, resulting in complicated and time-consuming analytical procedures [1,3]. In addition, they are destructive in nature, which limits their suitability for rapid screening during trading, transportation, retail, and consumption. Particularly in cold-chain circulation scenarios, conventional methods exhibit clear limitations in terms of timeliness and operational convenience [1,3,4].

In recent years, with the rapid advancement of machine vision and artificial intelligence technologies, deep learning-based nondestructive detection of food quality has gradually emerged as a major research focus [5,6]. Compared with methods such as spectroscopic techniques and electronic noses, image-based approaches offer distinct advantages in terms of equipment cost, operational complexity, and practical applicability [5,6,7]. In particular, the widespread availability of smartphone cameras can collect and analyze the appearance changes of food such as color and gloss at any time during storage [7,8,9], which provides a practical basis for the construction of low-cost, portable and field-deployable quality inspection systems [10,11,12,13,14,15]. Moreover, the development of deep learning has further enhanced the application potential of image recognition technologies in food quality evaluation. Conventional machine vision methods usually rely on manually engineered features, such as color histograms, texture descriptors, or threshold-based segmentation results [5,6]. However, these features are often sensitive to variations in illumination conditions, background interference, and individual sample differences, thereby limiting their generalization ability. In contrast, convolutional neural networks (CNNs) can automatically learn hierarchical feature representations from large numbers of samples and exhibit stronger modeling capability for complex backgrounds, nonlinear variations, and subtle appearance differences [5,6,7]. As a result, they have demonstrated considerable application value in areas such as meat freshness identification and aquatic product quality detection [8,9,16].

The application of image recognition methods to abalone freshness evaluation currently faces several challenges [17,18]. On the one hand, the abalone surface is often characterized by reflectance, moisture, and specular highlights, which can easily interfere with visual image acquisition. On the other hand, the appearance changes associated with the early stages of spoilage are relatively slow and subtle, making visual inspection by the naked eye highly subjective. In addition, inter-individual differences in size, color, and imaging posture further increase the difficulty of model learning and generalization. Compared with destructive chemical detection, smartphone vision requires no chemical reagents and realizes on-site rapid detection; against manual feature-based machine vision, ResNet50 can automatically extract subtle spoilage texture features of abalone; relative to lightweight networks and ViT, ResNet50 achieves more stable and balanced classification performance under complex surface reflection interference, and Grad-CAM further enhances the interpretability of prediction results. Therefore, developing a deep learning model that balances recognition performance, robustness, and interpretability is of great significance for improving the reliability of image-based abalone freshness detection [18,19,20]. In this study, smartphone images of refrigerated abalone were collected and labeled according to TVB-N measurements to build a high-robustness ResNet50 classification model with superior comprehensive performance, and Grad-CAM visualization was integrated to interpret the model’s decision logic. This study provides a low-cost, portable and interpretable technical basis for rapid nondestructive freshness assessment of abalone [17].

## 2. Materials and Methods

### 2.1. Materials and Chemicals

Magnesium oxide was purchased from Keyuan Biochemical Co., Ltd. (Shandong, China). Boric acid and bromocresol green indicator were obtained from Macklin Biochemical Co., Ltd. (Shanghai, China). Hydrochloric acid was purchased from Kunshan Jincheng Reagent Co., Ltd. (Kunshan, China). Methyl red indicator was obtained from Yuanye Bio-Technology Co., Ltd. (Shanghai, China). Ethanol (95%) was purchased from Xilong Scientific Co., Ltd. (Shantou, China). *Haliotis diversicolor* samples were purchased from Gongnong Market (Zhanjiang, China). An automatic Kjeldahl nitrogen analyzer was supplied by Foss Trading Co., Ltd. (Beijing, China).

### 2.2. Evaluation Metrics

The trained ResNet50 model was further evaluated using 772 test images, and its performance was assessed in terms of accuracy (Equation (1)), precision (Equation (2)), recall (Equation (3)), and *F*1*-score* (Equation (4)).

(1) $$Accuracy=\frac{TP+TN}{TP+TN+FP+FN}$$

(2) $$Precision=\frac{TP}{TP+FP}$$

(3) $$Recall=\frac{TP}{TP+FN}$$

(4) $$F1\;score=\frac{2\times Precison\times Recall}{Precison+Recall}$$

*TP* (true positive) denotes the number of positive samples correctly predicted as positive by the model. *FP* (false positive) represents the number of negative samples incorrectly predicted as positive. *FN* (false negative) refers to the number of positive samples incorrectly predicted as negative. *TN* (true negative) denotes the number of negative samples correctly predicted as negative.

### 2.3. Abalone Image Acquisition

To enhance the adaptability of the model to diverse environmental conditions, each abalone sample was placed on black background paper under natural lighting, and three images were randomly acquired from different heights and angles. Image acquisition was primarily conducted using the rear camera of a vivo smartphone (vivo X90), with an image resolution of 4032 × 3024 pixels. The abalone samples were stored at a refrigerated temperature of 4 °C, and each abalone was individually sealed in a sterile bag. Images were collected daily between 15:00 and 18:00 (Beijing time, China) throughout the storage period. Based on the TVB-N values measured on the corresponding day, the acquired images were labeled into two categories: fresh and spoiled [11,12,13,14,15,21,22]. A total of 1867 images were used in this study. Following the conventional dataset partitioning strategy for supervised deep learning classification, the images were randomly divided into training, validation, and test sets containing 900, 195, and 772 images, respectively. The test set comprising 772 images was strictly held out throughout model development and was not used for model selection, hyperparameter optimization, or selection of the best training epoch.

### 2.4. Determination of TVB-N

Three abalone samples were randomly collected each day for TVB-N determination. TVB-N was measured using an automatic Kjeldahl nitrogen analyzer according to the Chinese standard method (GB 5009.228-2016) [23] with slight modifications. Specifically, 6 g of the abalone sample was weighed and homogenized with 45 mL of distilled water. Then, 1 g of magnesium oxide was added and thoroughly mixed. The resulting mixture was distilled for 3 min, using 30 mL of boric acid solution (20 g/L) as the absorption solution. The mixed indicator was prepared from 1 g/L methyl red solution and 1 g/L bromocresol green solution at a volume ratio of 1:5. After distillation, the TVB-N value was calculated based on the volume of hydrochloric acid consumed in the titration.

Three biological replicates were analyzed per storage day to quantify TVB-N content. Under the controlled isothermal storage conditions (4 °C) with uniform initial sample quality, within-day variation in TVB-N was small, and three replicates were sufficient to represent the overall freshness status of the abalone batch at each time point. The daily mean TVB-N value was compared against the national standard spoilage threshold of 15 mg/100 g to assign binary freshness labels (fresh/spoiled) to all images acquired on the corresponding day.

### 2.5. Freshness Prediction Model

For abalone freshness classification, model selection should consider feature extraction capability, model complexity, and deployment feasibility. Several representative architectures, including MobileNet, ShuffleNet, DenseNet, ViT, ConvNeXt, and ResNet50, were evaluated under a unified experimental framework. ResNet50 was selected for subsequent analysis because it achieved the best overall performance in terms of accuracy, stability, and generalization [8,9,16,19].

Transfer learning was performed using a pretrained ResNet50 model within the PyTorch 2.14 framework. During training, the officially provided default pretrained weights were adopted, enabling the model to fully exploit generic visual features learned from the large-scale ImageNet dataset and thereby improving training stability and convergence efficiency under limited-sample conditions. On this basis, the original output layer designed for 1000-class classification was replaced with a fully connected layer for binary classification to discriminate between two abalone freshness states, namely fresh and spoiled. It should be noted that, in the present implementation, only the final fully connected layer of ResNet50 was modified, without introducing an additional Dropout module into the classification head. Therefore, the output layer essentially remained a standard linear classifier for binary classification.

**Figure 1:**
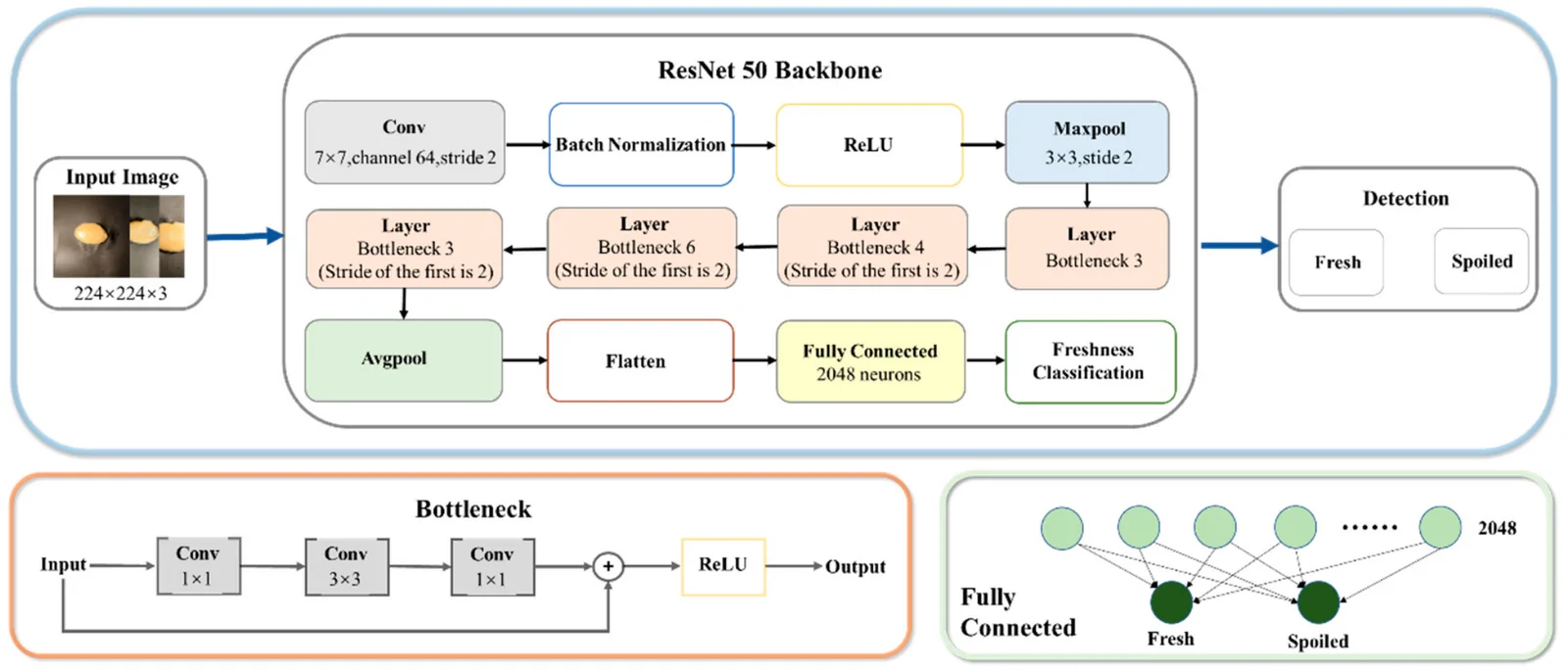
Model architecture flowchart.

In terms of network architecture, ResNet50 adopts a typical deep residual learning framework (See Figure 1). The input image is first processed by a 7 × 7 convolutional layer, followed by batch normalization and a ReLU activation function for initial feature extraction, and then subjected to max pooling for spatial downsampling. Subsequently, the feature maps are passed through four residual stages for hierarchical feature abstraction, with 3, 4, 6, and 3 residual units stacked in the respective stages, consistent with the standard ResNet50 architecture. The fundamental building block of the network is the Bottleneck residual unit, in which each block consists of 1 × 1, 3 × 3, and 1 × 1 convolutional layers. This design enables efficient feature learning through a dimensionality reduction–feature extraction–dimensionality restoration process, while cross-layer residual connections are established via identity mappings or downsampling branches, thereby effectively alleviating gradient vanishing and network degradation during the training of deep networks. After the final residual stage, adaptive global average pooling is applied to compress the high-dimensional feature maps into a one-dimensional representation, which is then fed into a fully connected layer to generate the final binary classification output.

All images were processed as three-channel RGB inputs. During training, random resized cropping (224 × 224) and horizontal flipping were applied for online augmentation, whereas validation images were resized to 256 pixels and center-cropped to 224 × 224. The images were then converted to tensors and normalized using the ImageNet mean and standard deviation.

For model training, cross-entropy loss was adopted as the objective function, and all trainable parameters were optimized using Adam with an initial learning rate of 1 × 10^−4^ and a batch size of 16. The learning rate was maintained constant throughout the 50 training epochs. After each epoch, the model was evaluated on the validation set using accuracy, precision, recall, and F1-score, and the parameter set achieving the highest validation accuracy was selected as the final optimal model. During inference, the predicted class was assigned according to the category corresponding to the maximum logit value.

To further investigate the basis of model decision-making and improve interpretability, Gradient-weighted Class Activation Mapping (Grad-CAM) was introduced during the inference stage for visualization analysis [19,20]. Unlike the common practice of directly selecting the final convolutional layer with a 7 × 7 spatial resolution as the target layer, this study employed the convolutional layer in layer2[-1] of ResNet50 as the Grad-CAM target. This design enabled the generation of heatmaps with higher spatial resolution and richer local details, thereby providing a more intuitive visualization of the key regions and important textural features on which the model relied for abalone freshness discrimination. The detailed parameter settings and corresponding modifications of the model are summarized in Table 1.

With respect to the experimental platform, model training was conducted on a high-performance computing server with an x86_64 architecture, equipped with dual Intel Xeon Platinum 8360Y processors providing a total of 144 logical processing units, and two NVIDIA GeForce RTX 4090 GPUs. Model construction and training were implemented using the PyTorch framework.

### 2.6. Visual Interpretation of the Model

Gradient-weighted Class Activation Mapping (Grad-CAM) is a technique that provides visual explanations for the decisions made by convolutional neural networks (CNNs) across a variety of models. This method generates coarse localization maps that highlight the regions of the input image contributing most substantially to the model’s decision, without requiring any modification of the network architecture or retraining of the model. The resulting heatmaps enable users to intuitively understand how the model operates and reaches its predictions, thereby enhancing confidence in model outputs for practical applications.

### 2.7. Statistical Analysis

All assays were conducted with three independent biological replicates (n = 3). Technical replicates within a single biological sample were not counted as independent observations and omitted from statistical analysis. Values are expressed as mean ± standard deviation (SD). All statistical analyses were performed in GraphPad Prism 9.0. Following confirmation of normality and equal variance assumptions, one-way analysis of variance (ANOVA) was applied for cross-group comparisons, followed by Fisher’s LSD post hoc test for pairwise analysis. A *p*-value < 0.05 was defined as statistically significant.

## 3. Results and Discussion

### 3.1. TVB-N Dynamics and Statistical Significance Defined the Spoilage Transition Point of Refrigerated Abalone

**Figure 2:**
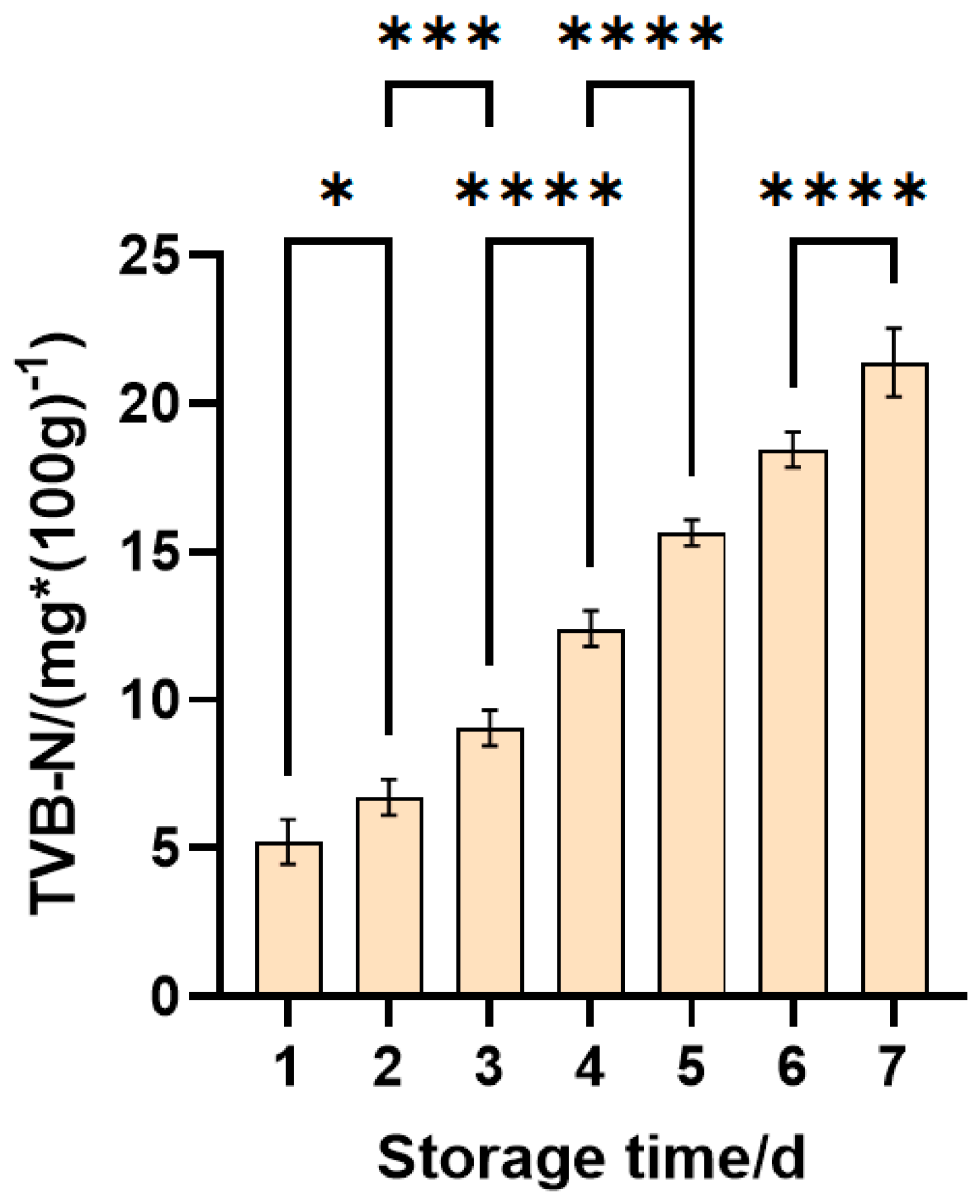
Total volatile basic nitrogen (TVB-N) content of abalone stored at 4 °C. Values are mean ± SD (n = 3 independent biological replicates per day). Asterisks indicate statistically significant differences between the indicated groups.

As shown in Figure 2, the TVB-N content of abalone exhibited a continuous upward trend during refrigerated storage at 4 °C, indicating progressive accumulation of volatile basic nitrogenous compounds and intensifying protein degradation and spoilage-related metabolic activity over time. Specifically, TVB-N values were 5.22 ± 0.75 mg/100 g on day 1, 6.71 ± 0.61 mg/100 g on day 2, 9.05 ± 0.60 mg/100 g on day 3, 12.41 ± 0.61 mg/100 g on day 4, 15.63 ± 0.43 mg/100 g on day 5, 18.43 ± 0.60 mg/100 g on day 6, and 21.37 ± 1.16 mg/100 g on day 7.

The difference between day 1 and day 2 was relatively small (*p* < 0.05), suggesting that the samples remained in a comparatively stable fresh state during the initial stage of storage. From day 2 onward, however, TVB-N increased progressively. On day 4, the value was still below the spoilage threshold of 15 mg/100 g, but already indicated that the samples were transitioning from the fresh stage toward quality deterioration. On day 5, TVB-N exceeded the established freshness limit for the first time, reaching15.63 ± 0.43 mg/100 g, marking a substantively meaningful deterioration point under the present storage conditions.

Significance analysis showed highly significant differences across the transitional period, particularly between days 2–3 (*p* < 0.01), 3–4 (*p* < 0.001), and 4–5 (*p* < 0.001), indicating that quality deterioration was not a purely gradual or random process, but rather a stage-dependent progression with a clearly identifiable turning point. In particular, the highly significant difference between day 4 and day 5 (*p* < 0.001) coincided with the crossing of the spoilage threshold, thereby providing strong statistical support for defining day 5 as the onset of spoilage.

Taken together, both the absolute TVB-N values and their statistical separation demonstrate that TVB-N was not only an effective chemical indicator of freshness loss in refrigerated abalone, but also a robust basis for label assignment in the image dataset. Therefore, defining samples from days 1–4 as fresh and those from days 5–7 as spoiled was scientifically justified, consistent with the national freshness evaluation criterion for aquatic products, and critical for establishing a reliable correspondence between external visual features and internal quality deterioration in subsequent deep learning-based classification.

### 3.2. ResNet50 Exhibited Stable Convergence and Favorable Generalization During Training

**Figure 3:**
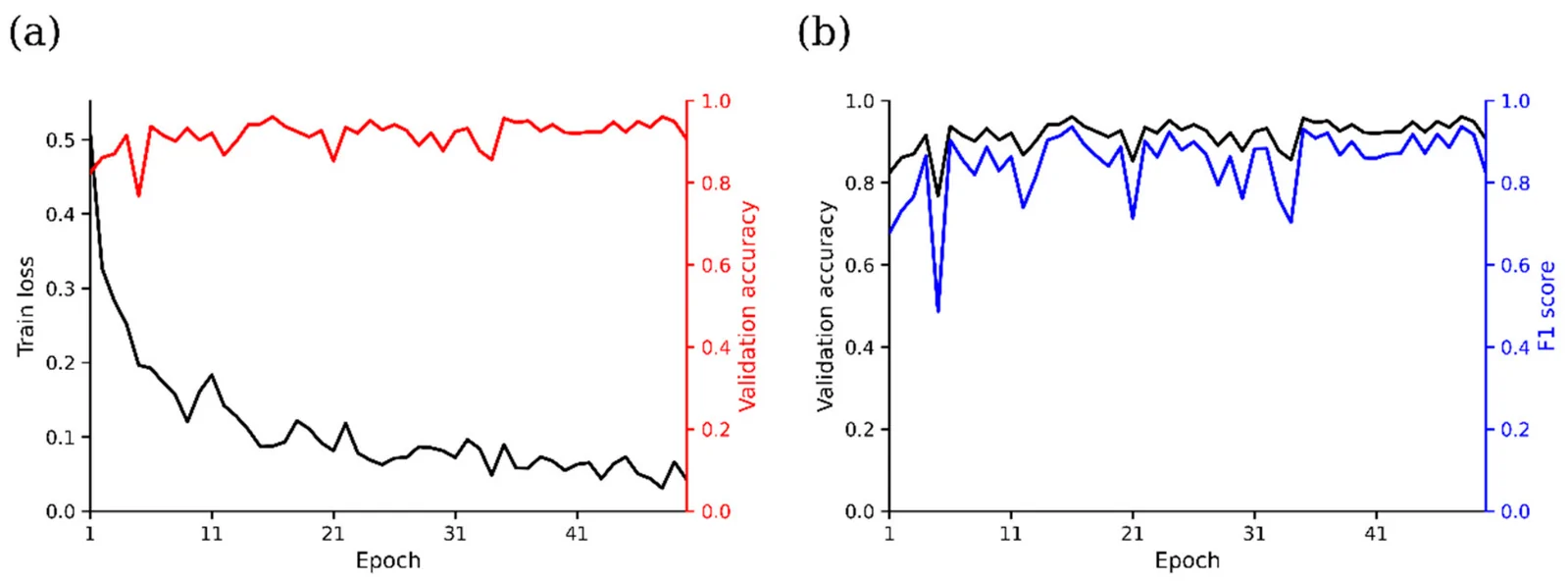
Classification performance of the ResNet50 model for abalone freshness classification: (**a**) changes in training loss and validation accuracy during training; (**b**) changes in validation accuracy and F1-score during training.

**Figure 4:**
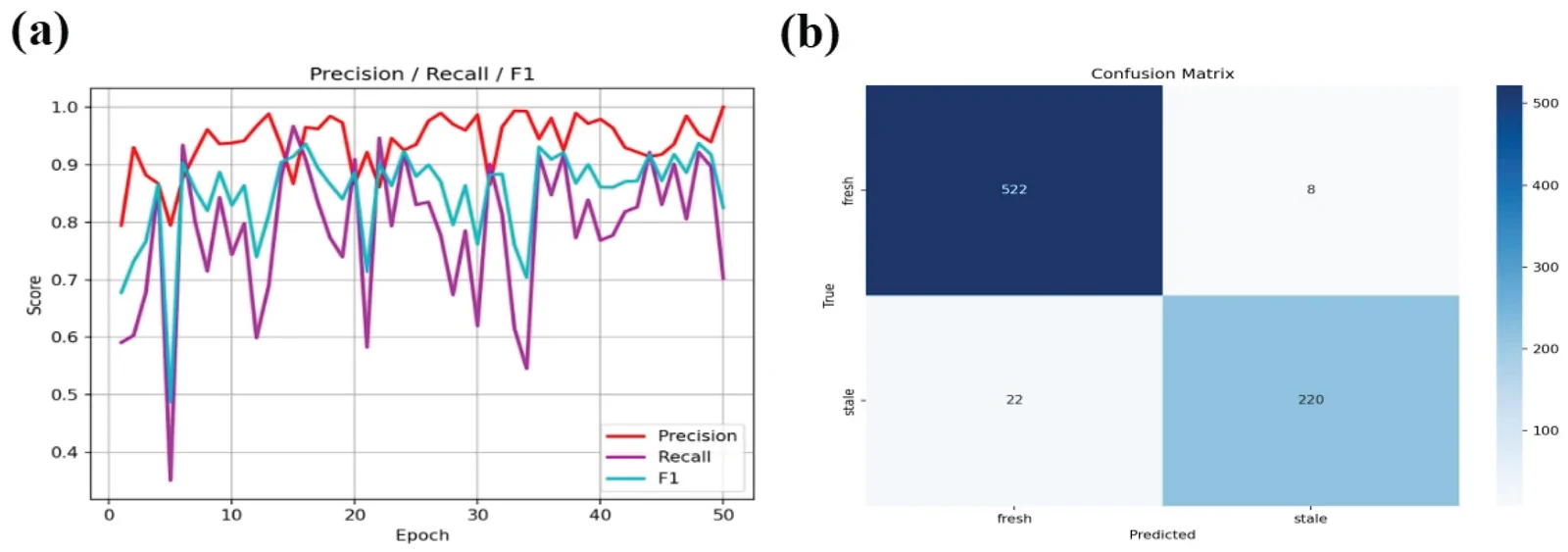
Training process and classification performance of the ResNet50 model for abalone freshness identification: (**a**) changes in precision, recall, and F1-score during model training; (**b**) confusion matrix of the final classification results for fresh and spoiled abalone samples.

Combined results from Figure 3 and Figure 4a show that ResNet50 converged efficiently during training. The training loss decreased rapidly in the early epochs and then stabilized at a low level, indicating that the transfer learning framework effectively captured visual features related to abalone freshness. Meanwhile, validation accuracy remained at a high level for most epochs, and the F1-score followed a trend like that of validation accuracy, suggesting balanced classification between fresh and spoiled samples with no obvious overfitting. Although recall fluctuated at several epochs, its overall level remained favorable, indicating robust sensitivity to spoiled samples and stable generalization ability.

Overall, this study developed a binary classification network for abalone freshness recognition based on the pretrained ResNet50 model provided by torchvision. By adapting the original classification head to the target task and integrating online data augmentation, cross-entropy loss, the Adam optimizer, and a multi-metric evaluation scheme, the classification performance and interpretability of the model for abalone freshness assessment were systematically investigated.

### 3.3. ResNet50 Provided the Most Balanced and Reliable Framework for Capturing Subtle Visual Deterioration Associated with Abalone Freshness Loss

**Figure 5:**
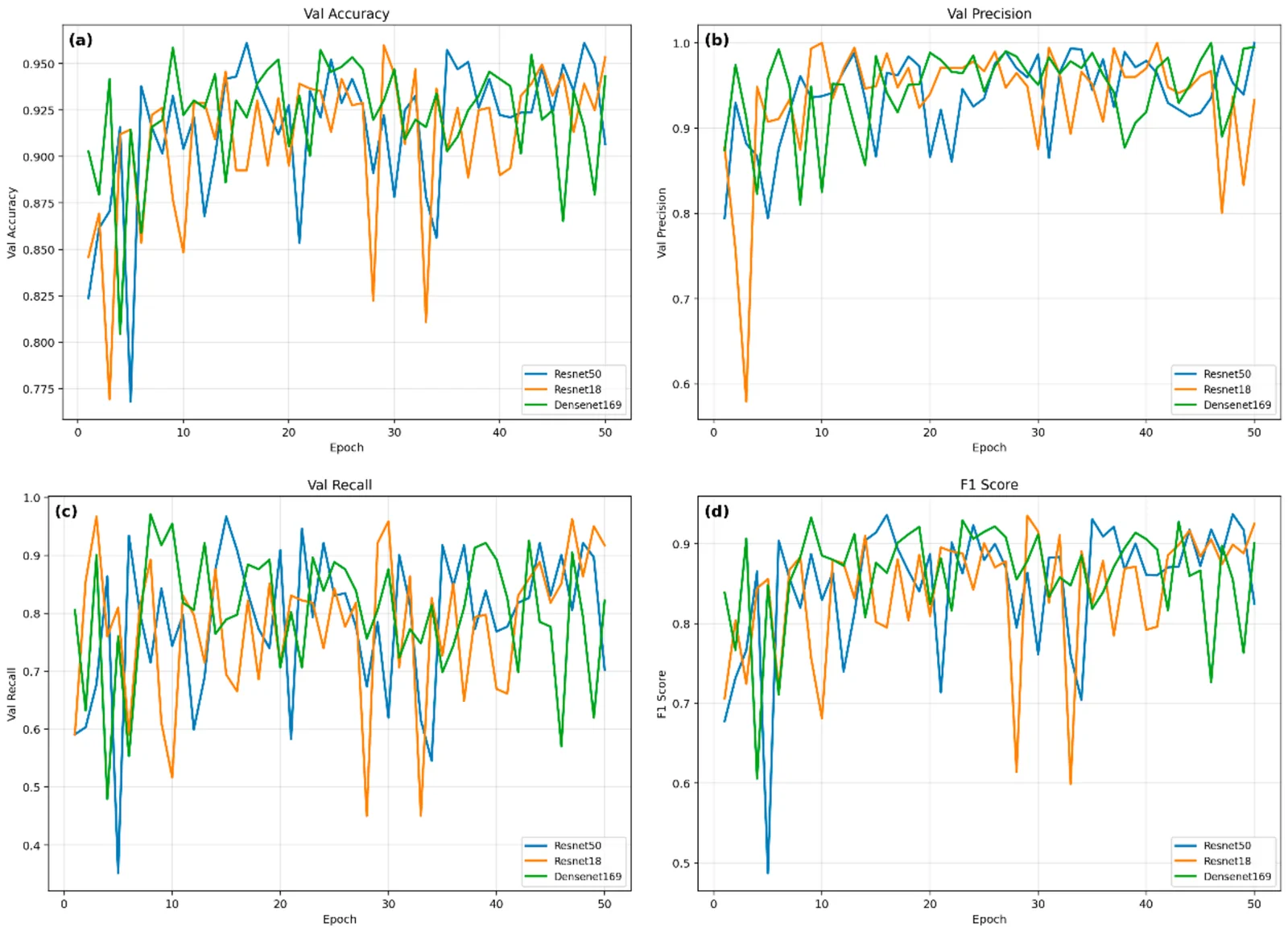
Per-epoch comparison of validation accuracy, precision, recall, and F1-score among the three top-performing models (ResNet50, ResNet18, and DenseNet169) for abalone freshness classification. (**a**) val accuracy of each epoch; (**b**) val precision of each epoch; (**c**) val recall of each epoch; (**d**) F1 score of each epoch.

**Figure 6:**
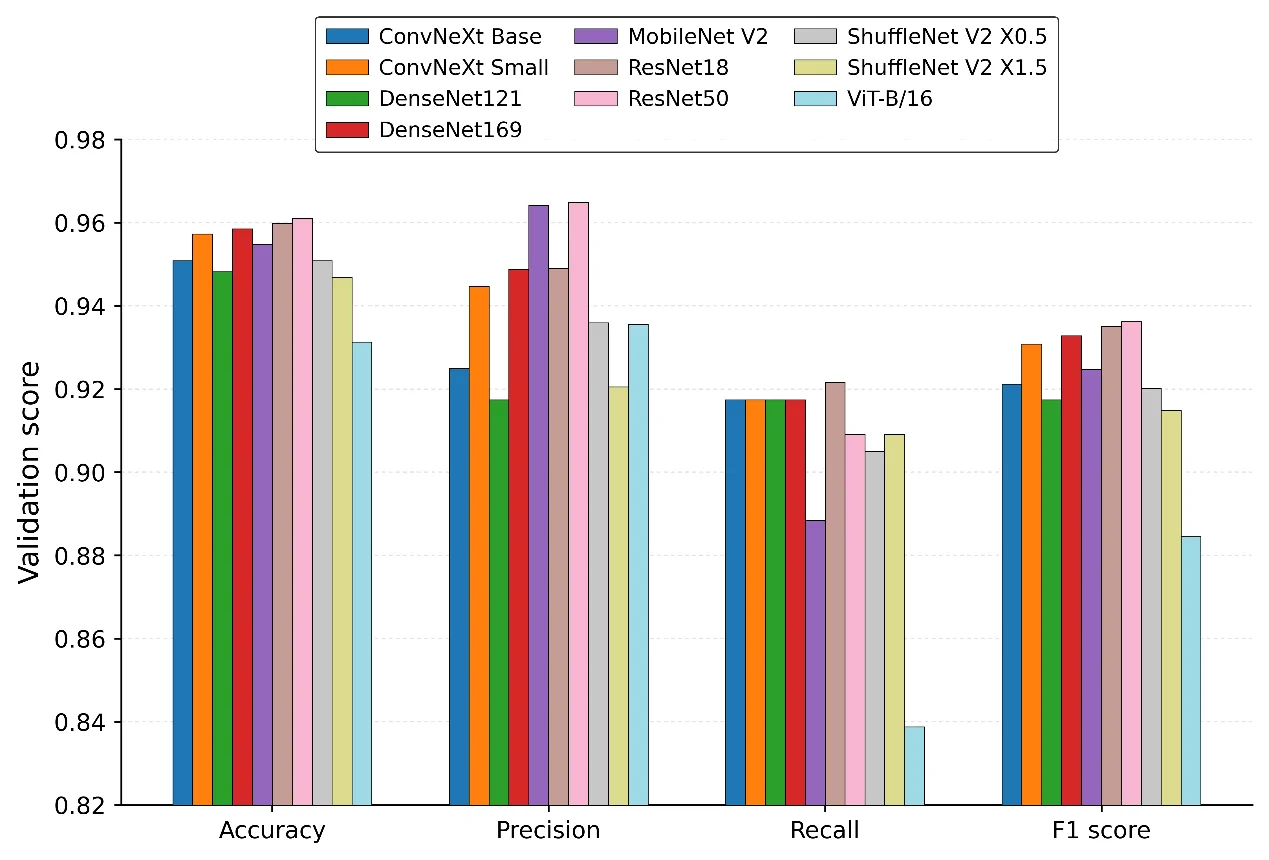
Comparative validation performance of different deep learning models for abalone freshness classification, expressed in terms of accuracy, precision, recall, and F1-score.

Model performance was evaluated using accuracy, precision, recall, and F1-score. Taken together, the results presented in Table 2, Figure 4, Figure 5 and Figure 6b indicate that ResNet50 achieved the highest observed validation accuracy (0.9611), precision (0.9649), and F1-score (0.9362), whereas ResNet18 achieved a slightly higher recall (0.9215). Notably, the F1-scores of ResNet50 and ResNet18 were very close (0.9362 vs. 0.9350). ResNet50 was selected for subsequent analysis because it provided the most favorable overall balance among the evaluated performance metrics under the current experimental setting.

By comparison, although ResNet18 yielded a slightly higher recall, suggesting a stronger tendency to identify spoiled samples, its lower precision and less stable performance implied that this increased sensitivity came at the cost of more erroneous predictions. DenseNet169 also showed competitive results; however, the per-epoch trajectories of the evaluation metrics demonstrated that ResNet50 exhibited smaller fluctuations during training, reflecting better convergence quality and more stable generalization behavior.

From the perspective of task characteristics, these performance differences are closely associated with the nature of visual cues involved in abalone freshness recognition. During refrigerated storage, the transition from fresh to spoiled is typically manifested by subtle appearance variations, including gloss attenuation, localized surface roughening, boundary alteration, and increased surface heterogeneity.

These changes are often fine-grained and can be easily confounded by biological variability, surface moisture, and specular reflection. Consequently, this task requires a model that is capable not only of deep feature extraction, but also of preserving local discriminative details while maintaining strong cross-layer semantic representation. The deep residual architecture of ResNet50 is well suited to this requirement, as residual connections facilitate gradient propagation, alleviate network degradation, and enhance hierarchical feature reuse, thereby improving the model’s ability to capture subtle spoilage-related visual patterns. This likely explains why ResNet50 outperformed several lightweight architectures as well as the Transformer-based model under the present experimental setting.

The confusion matrix further confirmed the practical discriminative effectiveness of ResNet50. Among the 772 test images, the model correctly identified 522 fresh and 220 spoiled samples, with only 8 fresh samples misclassified as spoiled and 22 spoiled samples misclassified as fresh, corresponding to an overall accuracy of 96.11%. These results suggest that the model exhibited relatively strong discriminative exclusivity toward the fresh class, meaning that it was less likely to incorrectly reject edible samples as spoiled. From an application perspective, such a characteristic is advantageous for reducing unnecessary economic losses caused by false alarms. Nevertheless, the presence of a small number of false negatives also indicates that further improvement is still needed in enhancing sensitivity to early spoilage-related visual changes, especially in practical scenarios where food safety warning is emphasized. Overall, the superiority of ResNet50 in this study can be attributed to its stronger capacity to model subtle appearance deterioration during refrigerated storage, making it the most suitable architecture for rapid and nondestructive abalone freshness classification under the current dataset and experimental conditions.

Previous studies have investigated seafood freshness using different sensing and modeling strategies. Lv et al. employed an electronic-tongue system combined with PCA and SVM for abalone freshness classification and reported a maximum classification accuracy of 95% after optimization of the sensor array [24]. Although this method enables effective freshness discrimination, it requires sample preparation, an electrode array, and electrochemical instrumentation. Rahman et al. applied multidimensional fluorescence imaging combined with PLSR to predict freshness-related K-values in frozen shrimp, achieving an R^2^ of 0.80 in independent validation [25]. This approach provides spatial information on freshness variation but depends on specialized fluorescence imaging equipment and optical components. Kumaravel et al. combined paper-based pH sensors with a Random Forest model for seafood freshness-related prediction and obtained low prediction errors across several seafood species; however [26], an additional colorimetric sensing element is required. In comparison, the present method directly analyzes RGB images acquired using a conventional smartphone without requiring additional chemical sensors, spectroscopic instruments, or electrochemical devices, and achieved an overall test accuracy of 96.11% for binary abalone freshness classification. Nevertheless, direct numerical comparison among these studies should be interpreted cautiously because they differ in target species, freshness indicators, prediction objectives, and evaluation metrics. The present method is also currently limited to binary freshness classification under the investigated acquisition conditions, and further validation under more diverse practical conditions is warranted.

### 3.4. The Spoilage Progression of Abalone Is Closely Associated with the Key Visual Features Extracted by Deep Learning

**Figure 7:**
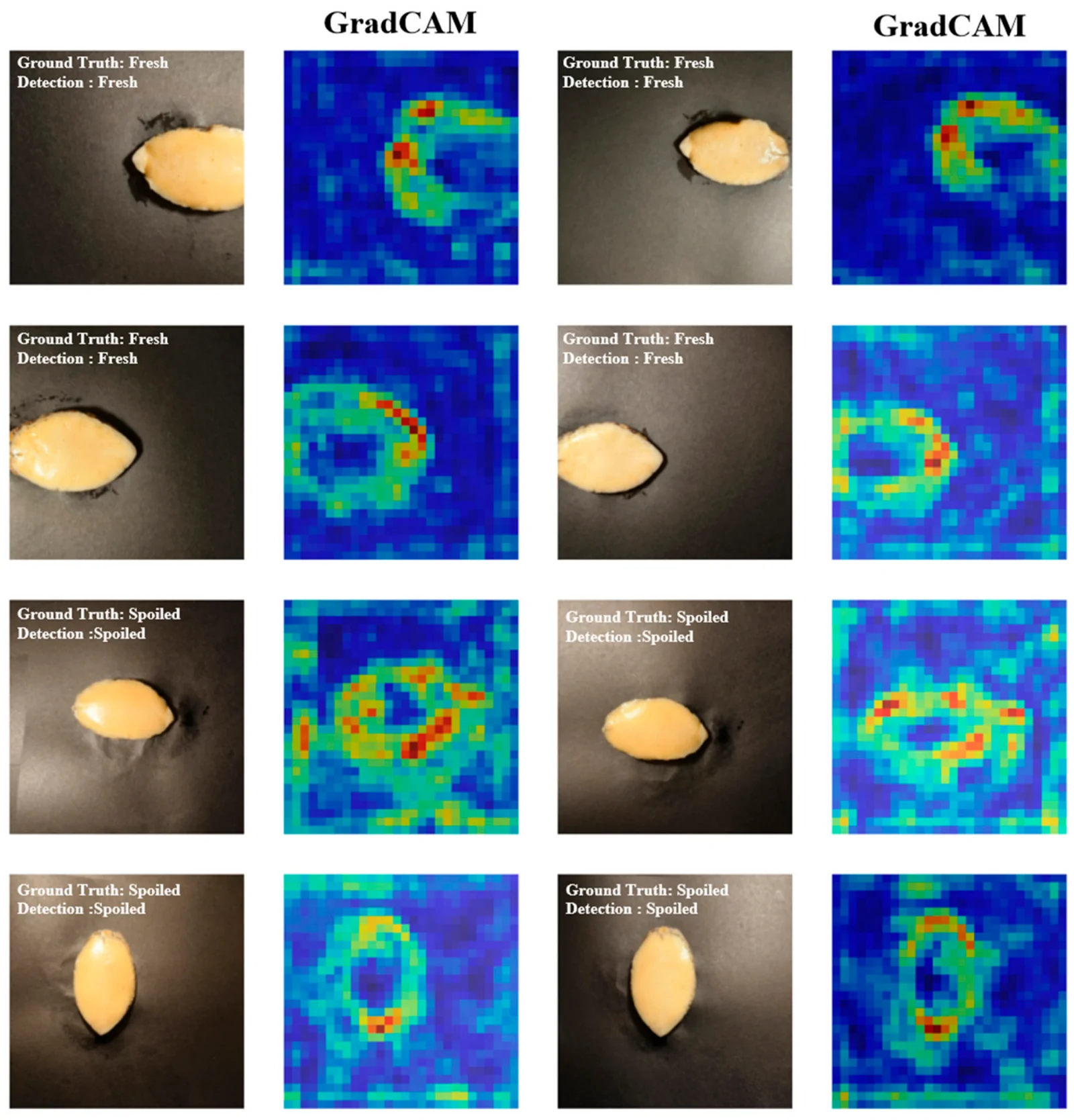
Grad-CAM visualization analysis of the ResNet50 model for abalone freshness identification. Representative classification results of fresh and spoiled samples and their corresponding Grad-CAM heatmaps are shown. The high-response regions were mainly concentrated on the abalone body and its marginal contour, indicating that the model primarily relied on key visual features associated with freshness changes for discrimination.

As shown in Figure 7, the Grad-CAM visualization revealed that the high-response regions of ResNet50 for both fresh and spoiled samples were predominantly concentrated on the edible body of the abalone and its marginal contour, rather than being diffusely distributed over the black background.

This observation indicates that the model predictions were primarily associated with visual information from the abalone body and marginal contour rather than with the black background. A more detailed examination of the class-specific activation patterns showed that, for fresh samples, relatively strong responses were frequently observed in regions with more homogeneous surface appearance and along the body contour. In contrast, spoiled samples tended to exhibit broader and more spatially heterogeneous activation patterns, particularly around surface and boundary regions. These differences suggest that the model utilized spatially distributed visual cues associated with the two TVB-N-defined freshness states. However, Grad-CAM only identifies image regions that contribute to model predictions and does not directly reveal the physicochemical or microstructural mechanisms underlying these visual differences. Therefore, specific processes such as moisture loss, tissue loosening, or microstructural alteration cannot be inferred from the present Grad-CAM analysis alone and would require independent physicochemical or structural measurements for confirmation. Overall, the Grad-CAM results provide visual evidence of the image regions contributing to ResNet50 classification and enhance the interpretability of the model without implying a direct mechanistic explanation of freshness deterioration.

To verify that the model discriminated freshness status rather than simply memorizing storage time, we further analyzed the classification behavior based on the obtained results. The model was trained for binary freshness classification anchored to the TVB-N spoilage threshold, not for regression of storage days. If the model had learned storage time patterns, predicted probabilities would show a gradual linear change across the 7-day storage period. Instead, the results showed a clear categorical separation around the spoilage threshold: samples from days 1–4 were consistently classified as fresh, while samples from days 5–7 were consistently classified as spoiled, with no gradual drift within each class. In addition, Grad-CAM visualization confirmed that the model focused on the abalone body and marginal contour where spoilage-associated appearance changes occurred, rather than on background or imaging artifacts that might co-vary with time. The model also showed consistent classification for individual samples with different sizes and initial appearances within the same freshness class, further supporting that it captured quality-related discriminative features rather than time-dependent trivial patterns.

## 4. Conclusions

In this study, a task-adapted deep learning framework was developed based on ResNet50 by integrating transfer learning with a tailored reconstruction of the classification head, enabling rapid and nondestructive abalone freshness discrimination using only smartphone-acquired images. Using 1867 abalone images collected under refrigerated storage at 4 °C and supervised labels assigned according to TVB-N measurements, the modified ResNet50 model effectively established the relationship between external appearance variation and internal quality deterioration. TVB-N increased progressively during storage and reached 15.63 ± 0.43 mg/100 g on day 5, exceeding the spoilage threshold; accordingly, samples from days 1–4 were defined as fresh and those from days 5–7 as spoiled, providing a reliable basis for supervised model training. Among the evaluated deep learning architectures, the task-optimized ResNet50 achieved the best overall performance, with a validation accuracy, precision, and F1-score of 0.9611, 0.9649, and 0.9362, respectively, and an overall test accuracy of 96.11%. Grad-CAM visualization further demonstrated that the model primarily relied on intrinsic visual cues closely associated with quality changes, particularly the abalone body and marginal contour, thereby supporting both the interpretability and decision reliability of the proposed approach. Overall, these findings demonstrate that the modified ResNet50 framework can effectively accomplish smartphone image-based abalone freshness recognition, highlighting its strong potential as a low-cost, portable, and nondestructive solution for digital quality evaluation and intelligent quality control of shellfish products during cold-chain circulation.

## Figures and Tables

**Table 1:** Detailed parameter settings of the adopted model.

No	Layer Name	Layer Size	Output Size
1	Image input	RGB image	224 × 224 × 3
2	Convolution 1	64 filters, 7 × 7, stride 2, padding 3	112 × 112 × 64
3	Batch normalization 1	BN	112 × 112 × 64
4	ReLU 1	-	112 × 112 × 64
5	Max pooling 1	3 × 3, stride 2, padding 1	56 × 56 × 64
6	Residual stage 1 (Layer1)	3 Bottleneck blocks	56 × 56 × 256
7	Residual stage 1–Block 1	1 × 1, 3 × 3, 1 × 1 + downsample	56 × 56 × 256
8	Residual stage 1–Block 2	1 × 1, 3 × 3, 1 × 1	56 × 56 × 256
9	Residual stage 1–Block 3	1 × 1, 3 × 3, 1 × 1	56 × 56 × 256
10	Residual stage 2 (Layer2)	4 Bottleneck blocks, first block stride 2	28 × 28 × 512
11	Residual stage 2–Block 1	1 × 1, 3 × 3, 1 × 1 + downsample	28 × 28 × 512
12	Residual stage 2–Block 2	1 × 1, 3 × 3, 1 × 1	28 × 28 × 512
13	Residual stage 2–Block 3	1 × 1, 3 × 3, 1 × 1	28 × 28 × 512
14	Residual stage 2–Block 4	1 × 1, 3 × 3, 1 × 1	28 × 28 × 512
15	Residual stage 3 (Layer3)	6 Bottleneck blocks, first block stride 2	14 × 14 × 1024
16	Residual stage 3–Block 1	1 × 1, 3 × 3, 1 × 1 + downsample	14 × 14 × 1024
17	Residual stage 3–Block 2	1 × 1, 3 × 3, 1 × 1	14 × 14 × 1024
18	Residual stage 3–Block 3	1 × 1, 3 × 3, 1 × 1	14 × 14 × 1024
19	Residual stage 3–Block 4	1 × 1, 3 × 3, 1 × 1	14 × 14 × 1024
20	Residual stage 3–Block 5	1 × 1, 3 × 3, 1 × 1	14 × 14 × 1024
21	Residual stage 3–Block 6	1 × 1, 3 × 3, 1 × 1	14 × 14 × 1024
22	Residual stage 4 (Layer4)	3 Bottleneck blocks, first block stride 2	7 × 7 × 2048
23	Residual stage 4–Block 1	1 × 1, 3 × 3, 1 × 1 + downsample	7 × 7 × 2048
24	Residual stage 4–Block 2	1 × 1, 3 × 3, 1 × 1	7 × 7 × 2048
25	Residual stage 4–Block 3	1 × 1, 3 × 3, 1 × 1	7 × 7 × 2048
26	Adaptive average pooling	AdaptiveAvgPool2d (1,1)	1 × 1 × 2048
27	Flatten	-	1 × 2048
28	Fully connected layer	Linear (2048, 2)	1 × 2
29	Output	Fresh/Spoiled	1 × 2

**Table 2:** Comparison of the best training epoch, training loss, and validation performance of different deep learning models for binary classification of abalone freshness.

Model	Best Epoch	Train Loss	Val Accuracy	Val Precision	Val Recall	F1-Score
Convnext Base	7	0.1962	0.9508	0.925	0.9174	0.9212
Convnext Small	47	0.0781	0.9573	0.9447	0.9174	0.9308
Densenet121	16	0.0987	0.9482	0.9174	0.9174	0.9174
Densenet169	9	0.1328	0.9585	0.9487	0.9174	0.9328
Mobilenet V2	25	0.0969	0.9547	0.9641	0.8884	0.9247
Resnet18	29	0.1016	0.9598	0.9489	**0.9215**	0.935
Resnet50	16	0.0874	**0.9611**	**0.9649**	0.9091	**0.9362**
Shufflenet V2 X0.5	31	0.1873	0.9508	0.9359	0.905	0.9202
Shufflenet V2 X1.5	29	0.0981	0.9469	0.9205	0.9091	0.9148
Vit B 16	37	0.1389	0.9313	0.9355	0.8388	0.8845

Bold text indicates the optimal result under the same metric.

## Data Availability

The original contributions presented in this study are included in the article. Further inquiries can be directed to the corresponding author.
